# Current Achievements in Flexible Piezoelectric Nanogenerators Based on Barium Titanate

**DOI:** 10.3390/nano13060988

**Published:** 2023-03-09

**Authors:** Olena Okhay, Alexander Tkach

**Affiliations:** 1 TEMA-Centre for Mechanical Technology and Automation, Department of Mechanical Engineering, University of Aveiro, 3810-193 Aveiro, Portugal; 2 LASI-Intelligent Systems Associate Laboratory, 4800-058 Guimaraes, Portugal; 3 CICECO-Aveiro Institute of Materials, Department of Materials and Ceramic Engineering, University of Aveiro, 3810-193 Aveiro, Portugal

**Keywords:** energy harvesting, piezoelectrics, nanomaterials, BaTiO_3_, polymers, composites, power output performance

## Abstract

Harvesting ambient mechanical energy at the nanometric scale holds great promise for powering small electronics and achieving self-powered electronic devices. The current review is focused on kinetic energy harvesters, particularly on flexible piezoelectric nanogenerators (p-NGs) based on barium titanate (BaTiO_3_) nanomaterials. p-NGs based on nanotubes, nanowires, nanofibres, nanoplatelets, nanocubes or nanoparticles of BaTiO_3_ fabricated in vertical or lateral orientation, as well as mixed composite structures, are overviewed here. The achievable power output level is shown to depend on the fabrication method, processing parameters and potential application conditions. Therefore, the most widely studied aspects, such as influence of geometry/orientation, BaTiO_3_ content, poling process and other factors in the output performance of p-NGs, are discussed. The current standing of BaTiO_3_-based p-NGs as possible candidates for various applications is summarized, and the issues that need to be addressed for realization of practical piezoelectric energy harvesting devices are discussed.

## 1. Introduction

Energy harvesting is attracting much attention nowadays, particularly if it is accompanied by high power density, simplicity, and miniaturization. Great advancements in low power integrated circuits, wireless communication and small electronics have reduced the demand for power consumption and increased the attractiveness of energy harvesting approaches. At the same time, the recent development of nanogenerators (NGs) has demonstrated a possible solution for the design of mobile electronics [1,2] and self-powered devices directly drawing energy from ambient sources [3,4]. NGs here refer to energy conversion systems containing materials at the nanometric scale.

The ambient sources suitable for energy harvesting are solar, temperature gradient, radio frequency, acoustic waves, and kinetic energy. Among these, kinetic energy, in the form of vibrations, random displacements, or forces, is ubiquitous and versatile in our ambient environment, including direct human activities such as walking, running, finger tapping, heartbeat and respiration, structural vibrations from industrial machinery, buildings, and transport vehicles, and fluid flows from tides, wind, geo-processes, etc. A number of research projects have been conducted to develop simple and efficient devices harvesting energy from vibrations by using piezoelectric materials. For random vibrations with frequencies from Hz to kHz, the available energy density is within the range of a few hundred microwatts to milliwatts per cubic or square centimetre [5,6,7]. Therefore, harvesting this type of energy offers great potential for remote/wireless sensing, charging batteries, and powering electronic devices. Moreover, energy harvesting from moving objects is a highly effective approach to solving energy problems.

NGs have opened a new gateway towards converting mechanical energy into electrical energy [8,9]. Piezoelectric, as well as triboelectric, NGs convert not only mechanical bending motions but also small movements of the human finger, heartbeat, and diaphragm activities into electrical signals [10,11]. Piezoelectric devices can produce a higher performance than triboelectric devices [12]. Piezoelectric bulk crystalline materials are widely used for applications, including transducers, sensors, and actuators, due to their affordability, stability, low cost of manufacturing, simple fabrication techniques, and the ability to be rendered into a variety of shapes [13], besides the fact that piezoelectricity shows good scaling with size [14]. However, their use in flexible devices is very limited due to their fragility. To overcome this problem, piezoelectric materials at the nanometric scale can be coated and/or mixed with flexible additives within composites, gaining flexibility at the cost of reduced piezoelectric response.

Such materials as lead zirconate titanate (Pb(Zr,Ti)O_3_, PZT), zinc oxide (ZnO), aluminium nitrate (AlN) and barium titanate (BaTiO_3_, BT) have been extensively studied in order to realize piezoelectric nanogenerators (p-NGs) [15,16,17,18]. Each of the considered materials can potentially satisfy the functional properties required for the fabrication of reusable, flexible, and conformable p-NGs. However, some differences can be appreciated, helping in the selection of the piezoelectric material to be used. By far the most distinctive element is piezoelectric behaviour, quantitatively represented by the piezoelectric coefficients (d_33_ and d_31_). As shown in Table 1, PZT has exceptional piezoelectric performance compared to other piezoelectric materials, being, e.g., employed by Wu et al. to fabricate flexible and wearable NGs, which can generate up to 200 mW/cm^3^ output power [19]. However, since PZT contains highly toxic lead, lead-free perovskite nanostructured piezoelectric materials have attracted attention for use in p-NGs [20]. At the same time, AlN and ZnO are lead-free, biocompatible, and non-ferroelectric materials showing piezoelectricity at a wide range of temperatures. However, despite both these materials possessing several advantages and similar properties, they have lower d_33_ and d_31_ values than those for BT. Moreover, BT is a valid non-toxic alternative to PZT, showing higher d_33_ than that for piezoelectric polymer polyvinylidene fluoride (PVDF) and its copolymers, polyvinylidene difluoride poly(vinylidene fluoride-co-trifluoro-ethylene) (P(VDF-TrFE)) and poly(vinylidene fluoride-co-hexafluoropropylene) (P(VDF-HFP)) [21].

The main mechanism of and studies on p-NGs with different basic materials, design, fabrication methods and generated power were first briefly overviewed by Kumar and Kim in 2011 [26]. Then, a number of review articles were published on different piezoelectric materials for mechanical energy harvesting (e.g., [27,28,29]), as well as on the current state of stretchable [30] or flexible p-NGs (e.g., [31]). However, p-NGs based on BaTiO_3_ were mentioned only superficially among energy harvesters fabricated using other piezoelectric materials. Only very recently were BaTiO_3_-based p-NGs separately overviewed and summarized with regard to their output voltage by Korkmaz and Kariper [32], since flexible p-NGs based on BaTiO_3_ have been widely studied over the last decade due to significant developments in robotics and interfaces between humans and machines. Here, we not only focus, in Section 2, on these studies with regard to the structures of the BaTiO_3_-based flexible p-NGs and the types of BT nanomaterials used, but also organise them into composites with non-piezoelectric and piezoelectric polymers in Section 3 and Section 4, respectively. Moreover, the effects of the concentration of used BT nanomaterials, poling process, applied mechanical stress mode parameters and p-NG thickness on the output voltage of flexible p-NGs are discussed in Section 5. Furthermore, since the output voltage is shown to be thickness-dependent, in contrast to the aforementioned review articles the reported results are summarised by power density in Section 6. Finally, some application examples are given in Section 7, followed by conclusions.

## 2. BaTiO_3_ Nanomaterial Types and Design of Flexible p-NGs Structures

Different types/forms of BT nanomaterials have been used for the fabrication of p-NGs, including ribbons of transferred film [17], nanoparticles (NPs) [33,34,35,36,37,38,39,40,41,42], nanowires (NWs) [43,44,45,46,47,48], nanofibres (NFs) [49], fibres made of NPs [50,51,52] or fibres made of NWs [45], nanotubes (NTs) [53,54], nano-cubes (NCs) [55,56], nanocrystals (NCr) [57], as well as the combination of different types of BT, such as the mixture of NWs and NPs [58].

**Figure 1:**
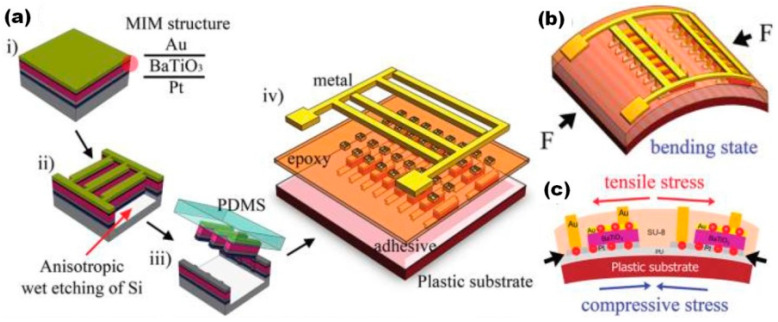
(**a**) Schematic illustration of the fabrication steps: (i) Deposition of an amorphous 300 nm thick BaTiO_3_ film on a Pt/Ti/SiO_2_/Si substrate by rf magnetron sputtering, with further rapid thermal annealing at 700 °C for crystallization. (ii) Inductive coupled plasma-reactive ion etching of metal-insulator-metal (MIM) structures (Au/BaTiO_3_/Pt layers) by chlorine gas, using an Al and plasma enhanced chemical vapor deposited-SiO_2_ (PEO) mask made with a narrow bridge pattern (300 μm × 50 μm). (iii) Transfer of the MIM structures onto a plastic substrate by PDMS stamp that was peeled away after transferring. (iv) Fabrication of self-powered flexible devices. The metal contact area was then opened by a standard photolithography process. When the nanogenerator was bent (corresponding to (**b**)), charges were generated in each MIM structure due to the tensile stress induced by the deflection of the device (corresponding to (**c**)). (Reprinted with permission from [17] Copyright 2010, American Chemical Society).

The first flexible piezoelectric nanogenerator on plastic substrate was reported by Park et al. in 2010 as 1350 metal-insulator-metal (MIM) structures made of transferred 300 nm thick BT film [17]. Output voltage up to ~1.0 V and current ~26 nA with power density ~7 mW/cm^3^ for periodical bending/unbending with a finger were obtained. However, the proposed method for p-NG fabrication was highly complicated. MIM ribbons (300 μm × 50 μm) from BT film were obtained by a plasma-reactive ion etching process and transferred onto flexible Kapton substrate using a polydimethylsiloxane (PDMS) stamp, followed by its peeling away (see Figure 1) [17]. Due to difficulties during the transferring process, there are only few reports on flexible p-NGs using BT films. Only Takahashi et al. reported the output power level of ~2.3 μW for a vibration frequency of 5 Hz generated by such p-NG [59].

According to the majority of reports, the piezoelectric layer in p-NGs includes BT nanomaterials as a main component of the composites, or at least as an individual piezoelectric layer covered/encapsulated by a polymer such as polyvinyl chloride (PVC) or PDMS [60]. This often includes plasticizers, helping to solve the fragility problem by adjustment of the hardness and viscoelasticity of composites. Thus, the piezoelectric layers in BT-based p-NGs can use BT nanomaterials (oriented vertically or horizontally) or a fully mixed structure (without preferable orientation).

**Figure 2:**
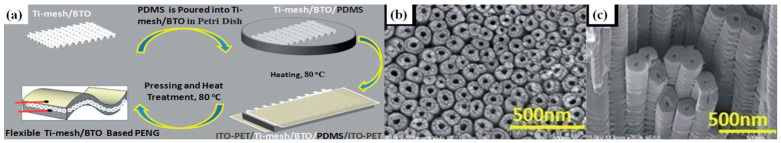
Schematic representation for the fabrication process of BT NT arrays on flexible Ti-mesh substrate (**a**). Surface (**b**) and cross-section (**c**) SEM image of BT nanotube arrays on Ti-mesh substrate after hydrothermal conversion of the TiO_2_ nanotube for 24 h. (Reproduced with permission of [53]. Copyright Royal Society of Chemistry, 2016).

Nanogenerators with vertical arrays between two horizontal electrodes are typically obtained using BT nanoparticles, with or without piezoelectric polymers. Tsege et al. reported vertically aligned (VA) BT NTs on Ti-mesh substrate, encapsulated by non-piezoelectric PDMS, as shown in Figure 2a, with the NT morphology presented in scanning electron microscopy (SEM) images, as shown in Figure 2b,c [53]. A maximum output voltage of 10.6 V and current of 1.1 µA were obtained with periodic bending at a frequency of ~0.7 Hz [53]. Similarly, a vertical BT NTs array was obtained after anodization of Ti foil, with a preparation of BT NPs by hydrothermal reaction, by Jeong et al., also encapsulated by PDMS [61]. However, such a device produced a significantly lower output voltage of ~150 mV and a current of ~3 nA by bending and releasing [61].

**Figure 3:**
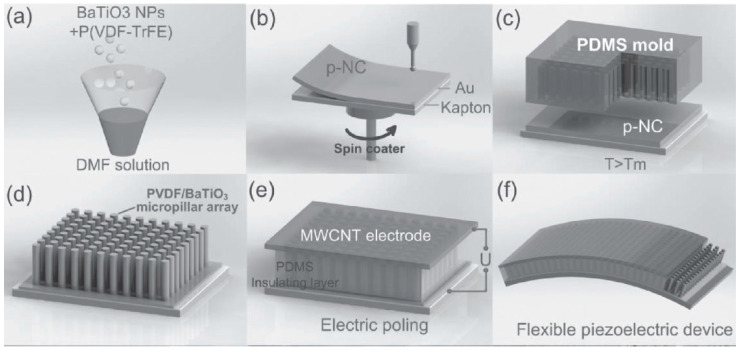
Experimental methods for fabrication of a high-performance piezoelectric nanogenerator based on a P(VDF-TrFE)/BT nanocomposite micropillar array, including solution mixing (**a**), spin-coating (**b**), moulding/hot pressing (**c**), annealing/mould removal (**d**) and PDMS covering/electroding (**e**). Schematic view of the flexible piezoelectric device (**f**). (Reproduced with permission of [40]. Copyright Wiley, 2017).

At the same time, micropillar arrays can be made from BT NPs mixed with piezoelectric polymer P(VDF-TrFE) formed inside the micropores of the soft PDMS mould by hot pressing, as reported by Chen et al., and can be seen in Figure 3 [40]. Here, a PDMS layer was also used for spin-coating onto the micropillar array acting as an insulation layer for electrical stability during the poling process and providing mechanical durability to the whole piezoelectric device. Moreover, Chen et al. used a multi-wall carbon nanotube (MWCNT) solution for the coating on the top of the surface. The maximal voltage and current outputs of fabricated p-NG could reach 13.2 V and 0.33 μA, respectively [40].

**Figure 4:**
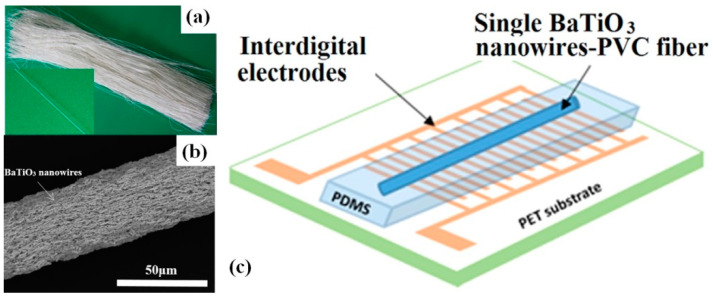
Digital images (**a**,**b**) and schematic of NG structure of <001> oriented BT nanowires–PVC composite microfibres (**c**). (Reproduced with permission of [44]. Copyright Elsevier, 2014).

In addition to vertically oriented BT arrays with parallel plate electrodes, a single horizontally oriented fibre obtained from BT NWs [48] or BT NWs-PVC [44] can be used, together with interdigital electrodes as p-NGs. As reported by Ni et al., a single BT NW with a diameter of less than 350 nm covered by PDMS can generate an output voltage of 0.21 V and an output current of 1.3 nA with periodical bending and releasing [48]. Similarly, Zhang et al. used a single fibre made of composite BT NWs-PVC (see Figure 4a–c) and reported an output voltage of 0.9 V and a current of up to 10.5 nA [44].

**Figure 5:**
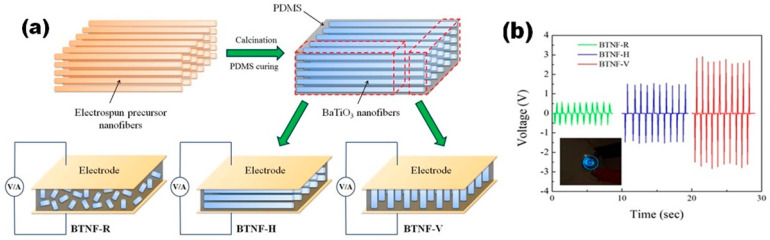
Schematic fabrication procedure of NGs based on BT nanofibres in three kinds of alignment modes within PDMS with piezoelectric test circuits (**a**). Output voltage changes of BT/PDMS p-NG under periodic mechanical compression (**b**). Inset in (**b**) is a photograph of a commercial blue light-emitting diode (LED) lit up by the electric energy generated from BT NF-V. (Reprinted with permission from [49]. Copyright 2016, American Chemical Society).

The difference between the vertical, horizontal and randomly-oriented types of structures was studied in depth by Yan et al. for flexible NGs with several alignments of BT NFs in the PDMS matrix (see Figure 5) [49]. For this purpose, PDMS was poured into uniaxially aligned calcined BT nanofibres. After penetration and curing of the PDMS, BT NFs–PDMS composite was cut into pieces along the aligned nanofibre direction or transverse direction to obtain horizontally-aligned BT NFs-based p-NGs (BT NF-*H*) or vertically-aligned BT NFs-based p-NGs (BT NF-*V*), respectively (see Figure 5a). For comparison, randomly-aligned BT NFs-based p-NGs (BT NF-*R*) were also fabricated with the same BT content, as represented in Figure 5. The highest average output voltage of ~2.67 V was obtained under applied pressure of 2 kPa for the p-NG with BT NF-*V*, in contrast to ~0.56 V and ~1.48 V measured for BT NF-*R* and BT NF-*H*, respectively (see Figure 5b). The highest average output voltage achieved for BT NF-*V* was explained by Yan et al. as due to three main reasons. First, a higher piezoelectric charge value can be delivered to the electrodes from the vertically-aligned BT nanofibres, due to the reduced number of polymer barriers compared to that for the two other composites. Second, fewer polymer barriers also make the poling of the vertically-aligned BT more efficient compared with that for the other two composites, where a significant value of the electric field has also to be applied to the polymer phase, which is not piezoelectrically active. Third, nanofibres connected vertically between electrodes are more compliant to mechanical stress [49].

Table 2 summarizes the features of p-NGs with strong vertical or lateral orientation of BaTiO_3_ nanomaterials, as reported by Yan et al. [49] and others [48,53]. Although a definitive conclusion cannot be made on composites with different geometrical parameters under dissimilar mechanical stimuli, the values of output voltage of p-NGs based on strongly vertically oriented BT nanomaterials are higher than those of p-NGs with horizontally oriented nanomaterials.

In contrast to the rarely reported p-NGs based on strongly vertically or horizontally oriented BT nanomaterials, p-NGs with randomly oriented BT nanomaterials are the most common in the literature. Typically, BT nanomaterials were mixed with non-piezoelectric additives such as resin [42], PDMS [37,41,49,54,55,57], or PVC [44] to increase flexibility, or combined with piezoelectric polymers such as PVDF [38,39,45,51,52,56], P(VDF-HFP) [33,34,35], P(VDF-TrFE) [46,50], or polylactic acid (PLA) [47] for enhancement of both flexibility and piezoelectric properties. However, the reported results mainly include only voltage and current values generated by p-NGs with different active areas and thicknesses, and with dissimilar applied stress modes and polling procedures. At the same time, power density (areal and volumetric), which is very important for the proper comparison of p-NGs and their final commercial application, is missing in many publications. Taking this into account, the aforementioned aspects will be discussed below, as far as possible, using the published data.

## 3. p-NGs Based on BaTiO_3_ Nanomaterials with Non-Piezoelectric Additives

There are several known combinations of BT materials with non-piezoelectric polymers for the fabrication of p-NGs on flexible substrates, such as polyethylene terephthalate (PET) or Kapton covered by a metallic or indium tin oxide (ITO) conductive layer. The most popular among them is the combination of PDMS and different BT nanomaterials such as nanoparticles [37,41], nanofibres [49], nanowires [43,48], nanowires together with nanoparticles [58], nanotubes [54], nanocrystals [57], and nanocubes [55]. Besides PDMS, other combinations with non-piezoelectric polymers such as p-NG based on fibre made of BT NWs with PVC [44], all-inkjet-printed NG made of BT NPs and resin [42], or polyacrylic acid (PAA) [36], were reported.

**Figure 6:**
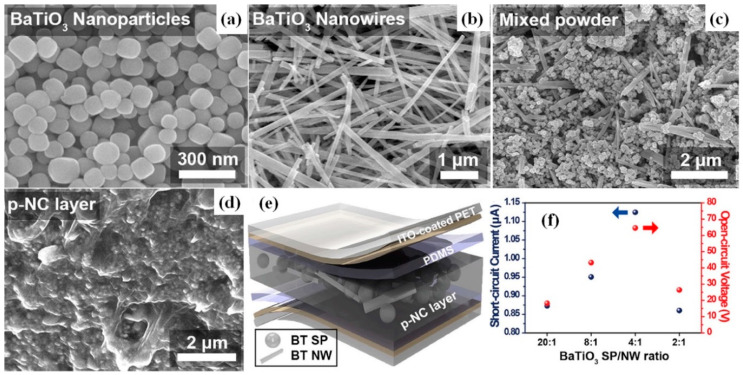
Hydrothermally synthesized BaTiO_3_ spherical nanoparticles (**a**) and nanowires (NWs) (**b**). Mixture of BT spherical NPs and NWs (**c**) and its embedded state in PDMS matrix (**d**). Schematic illustration of BT spherical NPs and NWs embedded in p-NG (**e**). The harvested electric signals generated from p-NG devices with different weights of BT SPs and NWs ratios (**f**). (Reproduced with permission of [58]. Copyright Elsevier, 2017).

A simple mixture of BT NPs and PDMS was used for the preparation of p-NG by Suo et al. [37]. A device with 100-μm-thick film of 20 wt.% BT in PDMS showed an output voltage ~14 V under periodic compressive force at 20 Hz [37]. At the same time, BT NWs mixed with PDMS by Park et al. reached an output voltage and current up to 7 V and 360 nA, respectively, under bending mode, and a power of 1.2 μW at 20 MΩ load resistance [43]. Moreover, Baek et al. studied p-NG, using simultaneously both BT NWs and NPs poured into a PDMS matrix (see Figure 6). This resulted in a high output voltage of ~60 V and the highest power of 40 μW at 500 MΩ for a piezoelectric composite with 4:1 ratio between spherical BT NPs (SPs) and NWs [58].

To obtain biodegradable and biocompatible nanogenerator BT, NPs were mixed with chitosan by Pongampai et al. [62]. Open circuit voltage (V_oc_) ~ 110.8 V and short circuit current (I_sc_) ~ 10 μA were obtained at an external force frequency of ~0.5 Hz and a magnitude of ~250 N for 3 × 3 cm^2^ BT NPs-chitosan p-NG with 160 μm thickness [62].

**Figure 7:**
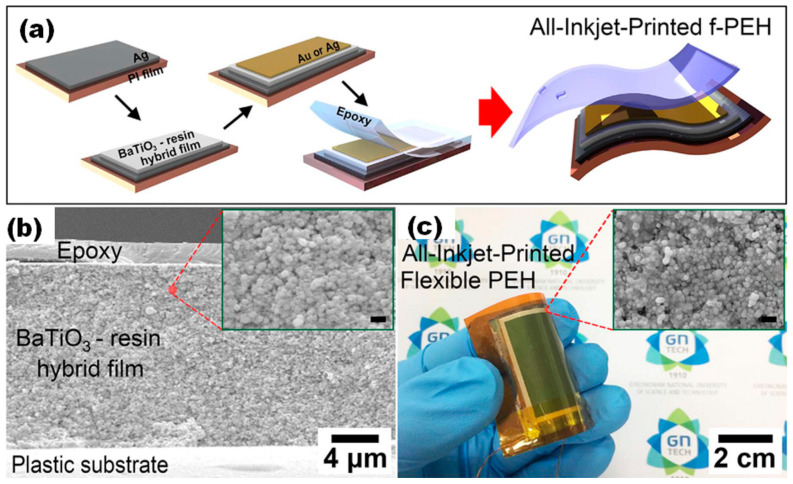
Scheme of the sequential process for the all-inkjet-printed f-PEH fabrication (**a**). The cross-sectional SEM images of all-inkjet-printed f-PEH (**b**). The inset shows the magnified cross-sectional image of a BaTiO3-resin hybrid film (scale bar: 500 nm). A photograph of the f-PEH with a sample size of 5 cm × 5 cm (activation area of 3 cm × 4 cm) achieved by inkjet-printing of piezoelectric hybrid film and conductive layers (**c**). The inset shows the top surface of an inkjet-printed piezoelectric hybrid film (scale bar: 500 nm). (Reproduced with permission of [42]. Copyright Elsevier, 2017).

Another method for the preparation of flexible p-NG or a piezoelectric energy harvester (f-PEH) was reported by Lim et al., who inkjet-printed BT NPs-resin hybrid layers on Ag coated polyimide (PI) (see Figure 7) [42]. V_oc_ and I_sc_ reached ~7 V and ~2.8 μA, respectively, under periodical bending by a programmable linear motor with a strain of 0.236% at a strain rate of 3.54%/s.

**Figure 8:**
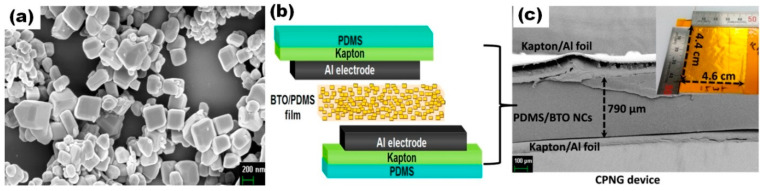
Field-emission SEM image showing highly crystalline BT NCs at 200 nm scale (**a**). Schematic drawing of composite p-NG device for harnessing mechanical energy (**b**). Cross-sectional SEM image of the p-NG device at the 100 μm scale; the inset is a photograph of the p-NG device (4.4 cm × 4.6 cm) without the PDMS packaging layer (**c**). (Reprinted with permission from [55]. Copyright 2018, American Chemical Society).

Besides BT particles without clear crystallographic orientation, there are reports on the use of oriented BT nanocrystals or polycrystals, as well as cube-shape nanoparticles in composites with polymers, for p-NG applications [36,55,57,63]. Yao et al. used 2D BT-oriented polycrystals in composite with PDMS and obtained an output voltage and current of 13 V and 200 nA, respectively, under a periodic bend-release mode [63]. Jeong et al. used untypical M13-virus as a template for the growth of BT nanocrystals [57]. The final BT NCr-M13 virus-PDMS structure p-NG represented I_sc_ and V_oc_ of ~300 nA and ~6 V, respectively, when bending and releasing [57]. Kim et al. reported on p-NG as an alternation of layers of cubic-like BT NPs stabilized by oleic acid (OA) ligands and PAA [36]. A 13-nm thick structure of 100 PAA-BT NCs bilayers generated an output voltage and current increasing from 0.4 V and 60 nA to 1.8 V and 700 nA, respectively, as the compressive force raised from 7 to 51 N without an additional poling process [36]. Moreover, both voltage and current were shown to drop along with decreasing bilayer number, and hence device thickness. In agreement with the later observation, the highest voltage value of this section, reaching 126.3 V generated at a constant mechanical pressure of ~0.001 MPa from the linear motor at a fixed acceleration of 1 m/s^2^, was reported by Alluri et al. for p-NG based on as thick as a 790 μm composite layer of BT NCs and PDMS, with the largest size reported here of BT nanomaterials used (nanocubes with a size up to 400 nm) (see Figure 8) [55].

Thus, once again a tendency of the output voltage to be enhanced by p-NG thickness prevents a proper comparison and definitive conclusion on composite effectiveness, based on the voltage values generated by composites with different geometrical parameters. At the same time, although composites of BT nanomaterials with PDMS were widely studied and different final parameters/output performances reported, their power density, particularly areal or volumetric, were not always provided. Even so, Table 3 summarises the main characteristics and parameters reported for BT-based flexible p-NGs with non-piezoelectric polymers. 

In addition, Park et al. studied BT NPs-PDMS composite in combination with carbon nanotubes (CNT), which resulted in much higher output voltage (~3.2 V) in comparison to BT NPs-PDMS with reduced graphene oxide (RGO) (~2 V) [41]. The low output voltage from the RGO-containing p-NG was explained by the geometrical difference between the CNT networks and RGO sheets.

## 4. p-NGs Based on BaTiO_3_ Nanomaterials with Piezoelectric Polymers

**Figure 9:**
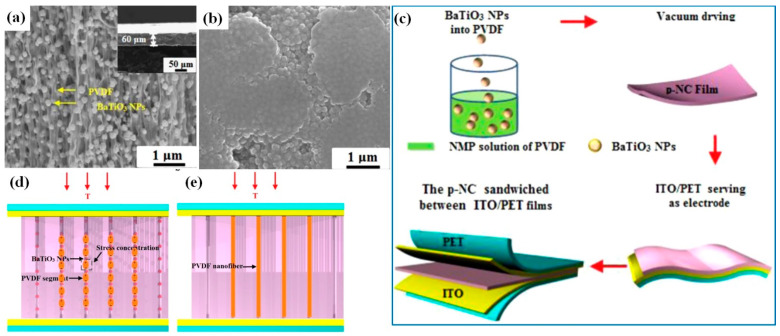
Side-view SEM image of the BT NPs-PVDF film (**a**). Inset in (**a**) is a low-magnification SEM image. Top-view SEM image of a piezo-nanocomposite film (**b**). Detailed fabrication procedure of flexible piezo-nanocomposite (**c**). Schematic diagram of the vertical stress applied to BT NPs-PVDF composite (**d**) and to pure PVDF (**e**). (Reproduced with permission of [38]. Copyright Elsevier, 2014).

Piezoelectric polymers are also considered as candidates for p-NGs owing to their remarkable flexibility and other mechanical properties. PVDF is their best-known representative, being a semicrystalline polymer, the *β* phase of which is characterised by a relatively high piezoelectric coefficient [64]. Therefore, polymers such as PVDF can also contribute to the generation of piezoelectric potential. In addition, compared to conventional PDMS, the PVDF solution can be of lower viscosity that is favourable for a more uniform distribution of BT NPs incorporated into PVDF matrix [38]. An output voltage as high as 150 V has been shown for 60-µm-thick p-NG made of BT NPs with PVDF by Zhao et al. [38]. This value is higher than the 126.3 V reported for 790-μm-thick composite of BT NCs and PDMS [55], but it was obtained at a four orders of magnitude higher pressure of 10 MPa, while a significantly lower output voltage of 35 V was reported in the case of 1 MPa applied pressure. Moreover, Zhao et al. used 150 nm large BT NPs (see Figure 9a,b) and a vacuum drying method (see Figure 9c) for the formation of an oriented fibre array of BT NPs-PVDF (see Figure 9a,d) [38]. Accordingly, PVDF fibres are separated into many segments by BaTiO_3_ nanoparticles that can act as stress concentration points when the film is subjected to a compression stress [38]. Therefore, the local deformation of the soft PVDF segments will be dramatically increased. However, there is no deformation enhancement in the pure PVDF film (Figure 9e), which results in a lower generated piezoelectric output of 15 V in comparison to that of 35 V for BT NPs-PVDF.

**Figure 10:**
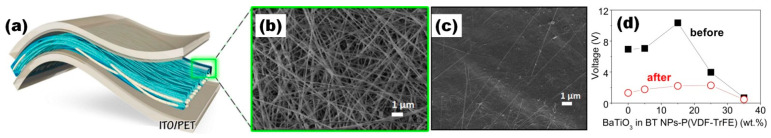
Schematic of the nanocomposite p-NG (**a**). Top-view image of electro-spun 35 wt.% BT-P(VDF-TrFE) nanocomposite nanofibers before (**b**) and after PDMS coating (**c**). (Reproduced with permission of [50]. Copyright Elsevier, 2016). Open-circuit voltage of BT NPs–P(VDF–TrFE) composite p-NGs before and after PDMS encapsulation as a function of BT amount (**d**) (adapted from [50]).

Like nanoparticles, BT nanocubes were also used for the fabrication of composite p-NGs with piezoelectric polymers. Alluri et al. used BT NCs for the preparation of BT NCs–PVDF composite that generated up to 7.99 V potential under a force of 11 N [56]. However, such output voltage was far from the highest voltage of 150 V under 10 MPa reported by Zhao et al. [38]. On the other hand, Siddiqui et al. reported a reducing effect for output voltage and enhanced flexibility after PDMS encapsulation of nanofibres prepared from BT NPs-P(VDF-TrFE) (Figure 10) [50]. Although the output voltage decreased from ~11 V to 3.4 V under tapping mode at 20 N after PDMS covering, the use of polymer is necessary because of the fragility of the BT-based nanomaterial [50].

**Figure 11:**
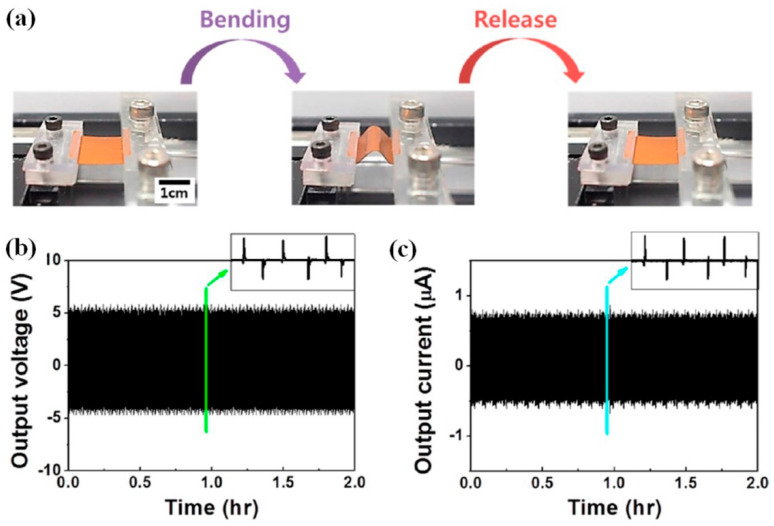
Optical images of the NG at bending and releasing state on bending stage (**a**). Cyclic measurement of open circuit voltages for 5400 cycles (**b**). The inset displays magnified signals. Cyclic measurement of short-circuit current for 5400 cycles (**c**). The inset shows magnified signals. (Reprinted with permission from [34]. Copyright 2014, American Chemical Society).

Moreover, voltage values up to 110 V were obtained at the applied pressure of 0.23 MPa to the surface by Shin et al., who studied the influence of the dimethyl-farmamide (DMF) and acetone solution on the dissolution of P(VDF-HFP) and particularly the influence of the covering area of BT NPs by piezopolymer on the output [34,35]. However, the output voltage was reported to be ~5 V only when this p-NG was studied at cycling bending stage (Figure 11) [34].

Another piezoelectric polymer, polylactic acid (PLA), with advantages such as biodegradable properties, was used by Malakooti et al. for the fabrication of p-NG by 3D printing [47]. However, reported output values of 1.4 V or 164.5 nA were not high [47].

Randomly oriented layer-by-layer (LbL) structures were reported by Yaqoob et al. as a combination of peeled-off BT NPs–PVDF films covered by graphene (Gr) [39]. The fabricated tri-layer p-NG showed a maximum output voltage of 10 V, along with a current of 2.5 μA, at an applied force of 2 N. The bi-layer structure generated just 2.7 V [39]. In addition to layered structures fabricated by a simple mixing of polymers with BT NPs, the NPs were also widely used for the fabrication of fibres, particularly by electrospinning. Such fibres made of BT NPs–PVDF or BT NPs–PVDF-graphene were reported by Lu et al. [52] and Shi et al. [51], respectively. The nanocomposite fibre of BT NPs–PVDF–graphene yielded an output voltage of ~11 V under a loading frequency of 2 Hz and a strain of 4 mm, higher than the V_oc_ of ~8 V for the composite without graphene [51]. Moreover, such a BT NPs–PVDF–graphene fibre p-NG had a maximum output power of 4.1 μW at 6.9 MΩ for 0.15 wt.% of graphene [51].

**Figure 12:**
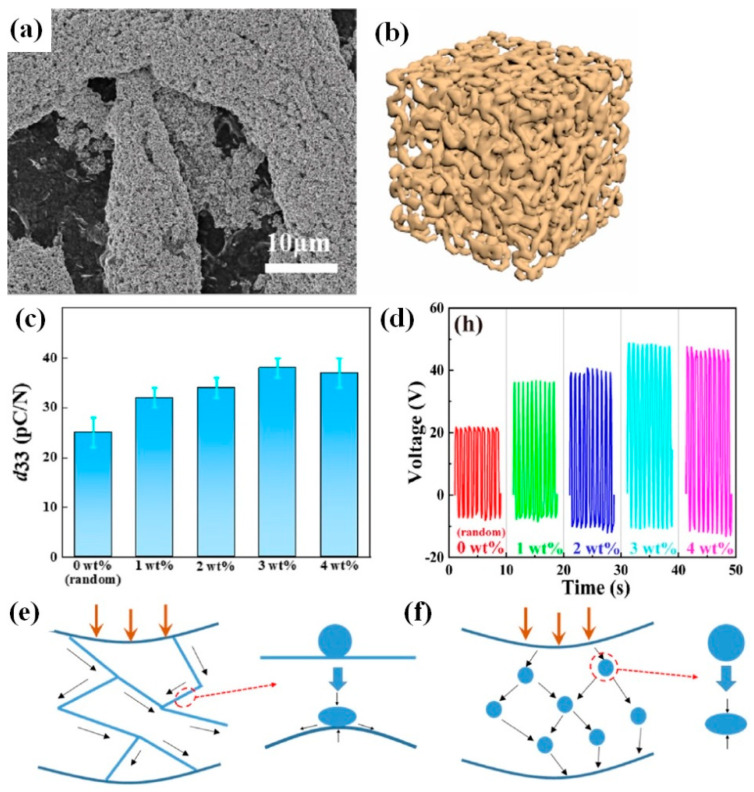
SEM image of the BT NP-impregnated cellulose scaffold after freeze-drying (**a**). The schematic diagram of the BT nanoparticles-impregnated cellulose scaffold (**b**). Piezoelectric coefficient of composite films with different methyl cellulose components added (**c**). Open-circuit voltage of PEHs with different cellulose content added. Open-circuit voltage of the PEHs with 3 wt.% methyl cellulose (**d**). Schematic diagram of forces transmitted to ceramic particles on a mesh scaffold (**e**). Schematic diagram of force conduction between ceramic particles through the matrix (**f**). (Reproduced with permission of [65]. Copyright Elsevier, 2022).

At the same time, methyl cellulose was successfully used as a supporting skeleton for BT NPs–PVDF–TrFE (Figure 12a,b) [65]. Young’s modulus and d_33_ (Figure 12c) were found to increase for freeze-dried cellulose mixed with BT NPs followed by pouring of PVDF-TrFE. As explained by Zhang et al., when stress is applied to the BT NPs–cellulose–PVDF–TrFE film, the stress transfer is more concentrated on the cellulose scaffold because the Young’s modulus of the cellulose scaffold is higher than that of the PVDF–TrFE matrix [65]. Thus, the deformation process of BT NPs fillers proceeds along with the deformation of the cellulose scaffold (Figure 12d). 

The net stress transferred to the piezoelectric BT nanoparticles on the scaffold is higher than that on typically distributed particles without a cellulose scaffold, as illustrated in Figure 12e. The stress between BT NPs is broadly transferred throughout the whole polymer matrix, and the mechanical conduction efficiency is poor due to a significant part of the stress being dissipated within the soft polymer matrix [65].

The main data for the related p-NGs on flexible PET/ITO, metallic or metalized PI, Kapton or PET, as well as polystyrene/Kapton/carbon-impregnated low-density polyethylene (PS/Kapton/C-LDPE) substrates, are summarized in Table 4.

## 5. Parameters Affecting Output Performance of p-NG

### 5.1. Concentration of BaTiO_3_ Nanomaterial in Composites

**Figure 13:**
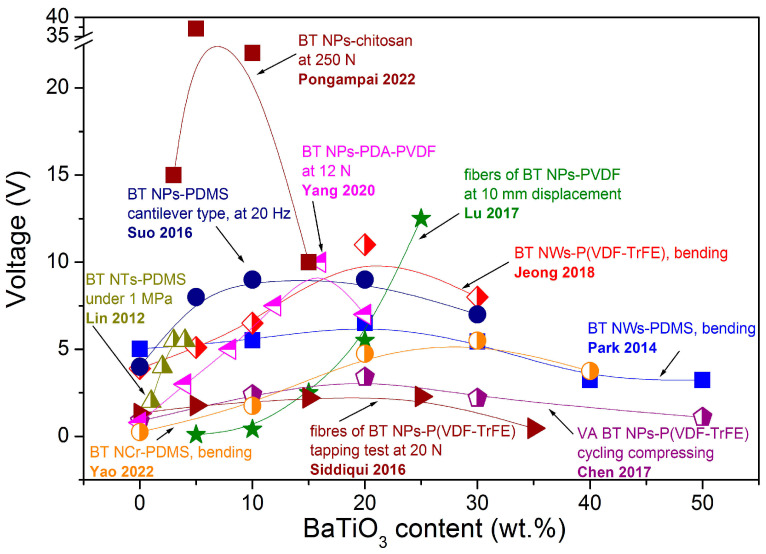
Output voltage values vs content of BaTiO_3_-based nanomaterials in flexible p-NGs (adapted from works by Suo et al. [37], Chen et al. [40], Park et al [43], Jeong et al. [46], Siddiqui et al. [50], Lu et al. [52], Lin et al. [54], Pongampai et al. [62], Yao et al. [63], Yang et al. [68]).

The fabrication process of the piezoelectric nanogenerator begins from choosing the type of BT materials and the ratio between the components in the composite. As already mentioned above, the highest reported output voltage of 150 V was obtained for an oriented fibre array structure of BT NPs-PVDF under 10 MPa, with 0.7 g of BT NPs and 0.3 g of PVDF powder (70 wt.% and 30 wt.%, respectively) by Zhao et al. [38]. Shin et al. also reported high output data of 75 V and 110 V for composites of 30 wt.% BT NPs and P(VDF–HFP) dissolved in acetone–DMF mixture, with ratios of 3:1 and 5:1, respectively [34,35]. However, neither Zhao et al. [38] nor Shin et al. [34,35] showed further data for p-NGs with a higher or lower BT amount in their comparison and used only one selected BT amount and only the main part of the available reports. At the same time, there are several reports on the systematic study of the BT concentration effect on the output voltages of p-NGs based on BT materials (summarized in Figure 13). According to Figure 13, the increase in the output voltage with BT content is always present for concentrations of different BT nanomaterials up to 5–30%, but further increase in concentration decreases the output. Moreover, there is no strong tendency related to the type of BT nanomaterials or the type of used polymers. At the same time, Chen et al. explained the decrease in the output in vertical arrays made of BT NPs–P(VD–TrFE) composite observed for BT concentrations higher than 20 wt.% by the agglomeration of BT NPs, which leads to the formation of cracks in the nanocomposite films and further adversely affects the performance of the nanogenerator [40].

### 5.2. Poling Process

**Figure 14:**
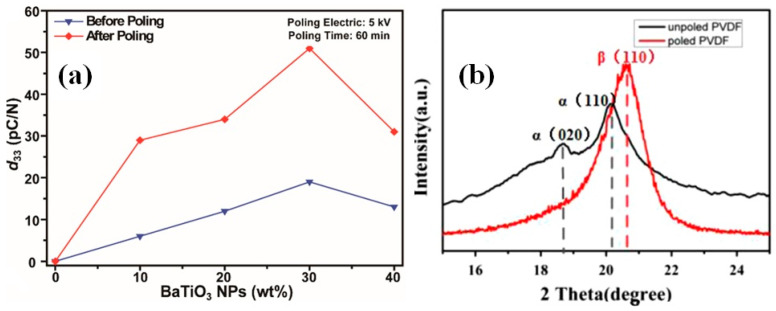
Piezoelectric coefficient (d_33_) of composite BT-PDMS films with BT contents of 10, 20, 30, and 40 wt.% obtained before and after poling (**a**). (Reprinted with permission from [63]. Copyright 2022, American Chemical Society). X-ray diffraction pattern of the poled and unpoled PVDF film (**b**). (Reproduced with permission of [38]. Copyright Elsevier, 2014).

For enhanced piezoelectric, all the dipoles of piezoelectric material need to be oriented in the direction of the field and for is, a poling process in particular is widely used before the characterization of p-NGs. As can be seen in Figure 14a, the piezoelectric coefficient d_33_ of the composite BT-PDMS films significantly increased after the poling process for all studied BT concentrations between 10 and 40 wt.% [63]. Moreover, in cases using piezoelectric PVDF polymer together with BT nanomaterials, the poling process is quite important, because during the poling the extent of the most piezoelectrically active *β*-phase of PVDF significantly increased, as reported by Zhao et al. (Figure 14b) [38], and has strong influence on the output results [70].

However, not all p-NGs overviewed were electrically poled before the output measurements (more details of the poling process, including applied field, temperature, and time, for the p-NGs described are summarized in Table A1 in Appendix A). Kim et al. studied the multi-layered structure of OA-BT NPs–PAA without an electrical poling process, postulating that the unpoled dipoles within the ferroelectric film can be aligned by the application of an external stress instead of an electric field [36]. Thus, an output voltage of 1.8 V was obtained using a compressive force of 51 N [36]. In addition, Siddiqui et al. [50], Shi et al. [51] and Guo et al. [45] reported the absence of a necessity to perform the poling process for p-NGs fabricated using the self-poled nanocomposite fibres obtained by electrospinning of BT NPs-P(VFD–TrFE), BT NPs–PVDF with graphene and BT NWs–PVDF, respectively. In this case, the high electrical field is already present during the fabrication process. Moreover, Shi et al. reported that, under the in-situ poling processes, the conductive graphene in BT NPs–PVDF can enlarge the local electric field and generate charges, thus resulting in a stronger Coulomb force, which can attract PVDF chains to crystallize into the *β* phase on the graphene surface [51]. This leads to an increased amount of *β* phase in the nanocomposites in comparison to pure PVDF nanofibres [51].

On the other hand, when the poling was performed, the electric field, time, and temperature values varied across a wide range:

- applied electric field was set to 400 kV/cm [46];
- time of poling varied up to 24 h [52,53];
- electric field was applied from room temperature up to 150 °C [41].

**Figure 15:**
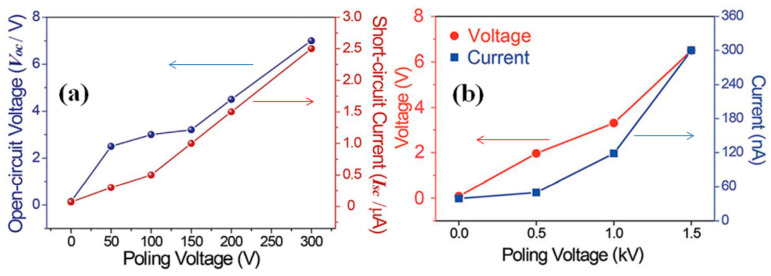
The output performance of BT NPs with resin (**a**) (Reproduced with permission of [42]. Copyright Elsevier, 2017) BT NWs with PDMS (**b**) (Reproduced with permission of [43]. Copyright Royal Society of Chemistry, 2014) as a function of the poling voltage.

According to our knowledge, there are no available reports concerning the time or temperature effect on output performance of p-NGs based on BT nanomaterials. However, the effect of the applied electric field during the poling process on the output voltage and current was demonstrated in many articles for different BT nanomaterials with different additives. For example, Figure 15a,b present such variations for BT NPs with resin [42] and BT NWs with PDMS [43], respectively. The output increases along with poling voltage for both systems.

Moreover, a general conclusion that a much smaller output was observed for unpoled p-NGs compared to those after the poling process can be made, based on several reports summarized in Table 5. It is obvious that the output voltage can be increased up to 25 times, from 0.2 V to 5 V, after poling, as was reported by Yan et al. [49].

### 5.3. Influence of Applied Mechanical Stress Mode

**Figure 16:**
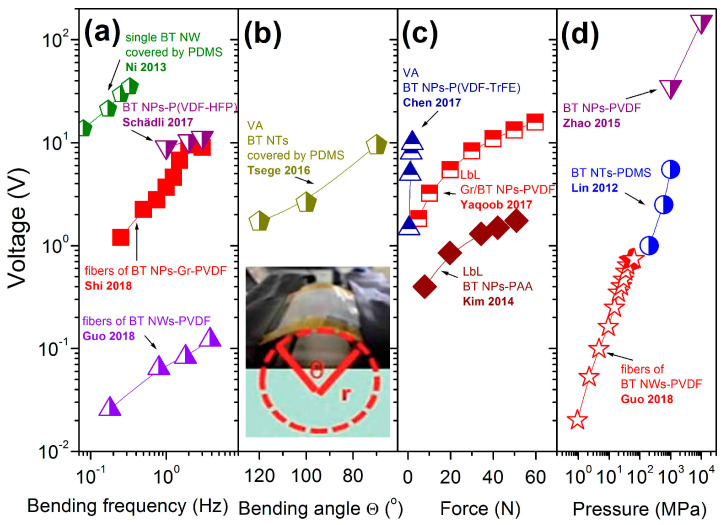
Dependence of the output voltage on bending frequency (**a**) (adapted from works by Schädli et al. [33], Guo et al. [45], Ni et al. [48], Shi et al. [51]), on bending angle (**b**) (the inset in (**b**) shows the image of the possible bending angle, adapted from work by Tsege et al. [53]), on mechanical force (**c**) (adapted from works by Kim et al. [36], Yaqoob et al. [39], Chen et al. [40], and on applied pressure (**d**) (adapted from works by Zhao et al. [38], Guo et al. [45], Lin et al. [54]), for BT-based flexible p-NGs.

During the measurements, the output voltage and current of the piezoelectric nanogenerators appeared as a response to applied mechanical stress. In the simplest manner, this can be done by a simple bending/releasing of p-NG by fingers, or by a special device such as the one shown in Figure 13a. The performance of the nanogenerator is found to be very sensitive to the bending parameters, such as amplitude, stress/strain, bending angle, etc. However, many reports did not include these details, while some of them used dissimilar terminology that can produce different meaning for such measurements. At the same time, there are reports on the output voltage dependency on strain/bending displacement/bending amplitudes/angular bending curvature, mainly for fibre- or wire-based p-NGs with different additives. Moreover, for such p-NGs, the output voltage increases only up to some specific value of mechanical deformation and decreases after that, as observed for fibre made of BT NPs [51,52], or for BT NW [43,48]. In addition, the output response depends on bending frequency, bending radius, and bending angle. Figure 16a depicts that voltage increases with bending frequency, at least up to some limit, as shown by Shi et al. for fibre p-NGs based on BT NPs–PVDF [51]. The relationship between the output voltage and bending angle was presented by Tsege et al. for vertically aligned BT NTs covered by PDMS [53]. As shown in Figure 16b, the output signal increases along with decrease in bending angle.

Some authors mentioned the values of force (see Figure 16c) or external pressure (see Figure 16d) applied to the studied p-NGs, both of which increased the output voltage. However, both weak and strong dependences of voltage on applied force can be seen in Figure 16c, comparing a 200 μm thick layered structure of NPs–PAA [36] with a layered but thinner, and thereby more flexible, 60 μm thick structure of NPs–PVDF with graphene layer [39]. However, Zhao et al. reported the voltage increasing from 35 V to the highest point at 150 V along with applied pressure from 1 MPa to 10 MPa, respectively, for p-NGs based on BT NPs–PVDF (Figure 16d) [38]. Thus, output voltage as well as current is strongly dependent on applied external mechanical bending, force or pressure.

### 5.4. Thickness of Working Layer

**Figure 17:**
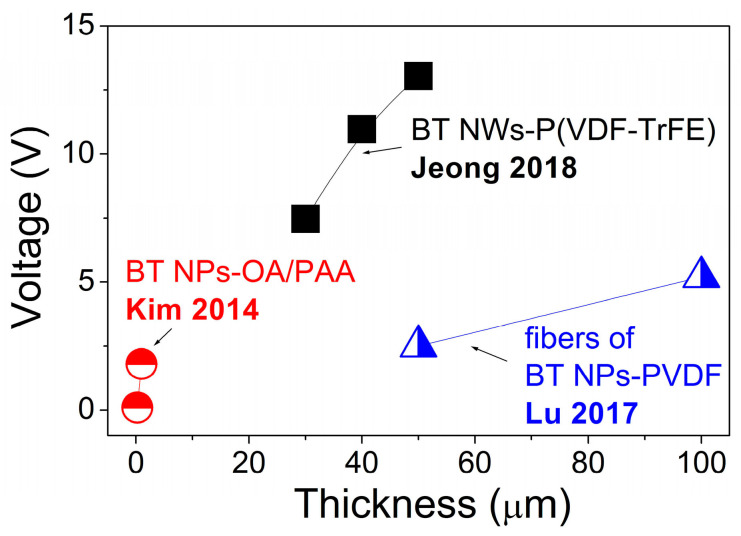
Relationship between the output voltage and thickness of flexible BT-based piezoelectric composites (adapted from works by Kim et al. [36], Jeong et al. [46], Lu et al. [52]).

Despite that only nanometre-scale materials are used for the fabrication of p-NGs, the thickness of the final device, as well as of the working BT-based composite layer, can be much larger. The increase of the output voltage along with thickness was demonstrated for composite fibres fabricated using BT NPs with PDMS [52], as well as for BT NWs with P(VDF–TrFE) [46] or for OA–BT NPs on PAA prepared as a multilayer structure [36], as shown in Figure 17. Moreover, as already seen in Table 3 and Table 4, one of the highest output voltages of 126.3 V among those presented here was reported for the p-NG with the thickest composite BT NCs–PDMS layers of 790 μm [55].

At the same time, the thickness of BT NWs–P(VDF-TrFE) layers over ~50 μm was found by Jeong et al. to significantly decrease the flexibility of p-NG [46]. Furthermore, if not very high output voltage but high-power density is aimed for in the p-NG, then not very thick, but rather thin film can be necessary, as will be discussed in the next section.

## 6. Power Performance

Having considered numerous reports on p-NGs fabricated using BT nanomaterials (presented in Table 3 and Table 4), often only open-circuit voltage V_oc_ and short-circuit current I_sc,_ obtained under repeatedly applied bending or force or displacement, etc., are presented as the p-NG output. However, power or power density values are necessary for proper comparison of the composite effectiveness for p-NG applications, and these values cannot be obtained as a product of V_oc_ and I_sc_, which are measured at extremely different load resistances. Therefore, besides the different mechanical conditions such as bending or force or displacement amplitudes and frequencies, the p-NG output power depends also on the load resistance. Then, the output power can be calculated based on Equation (1):

(1) $$P=\frac{1}{T}\int^{\;}\frac{U^{2}\left(t\right)}{R}dt$$

where *U*^2^(*t*) is the square of the real-time voltage on the external load, *R* is the impedance of the external load (usually in MΩ), and *T* is the period of the pressing (or bending) and releasing [49]. Alternatively, the product of voltage and current measured on the same external load resistance provides the effective power for the fabricated flexible p-NG. Such power or power density values obtained or optimised at specified load resistance are summarized in Table 6, ordered by load resistance value.

**Figure 18:**
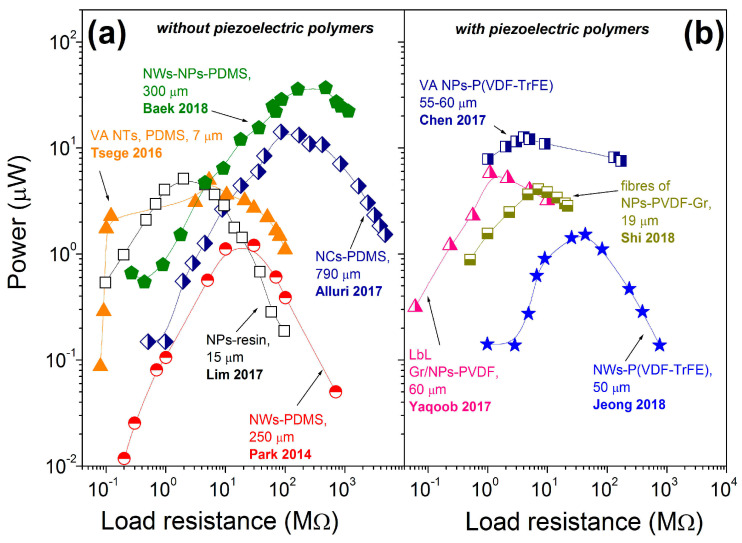
Power as a function of external load resistance reported for flexible p-NGs based on composited including BT nanomaterials with (**a**) non-piezoelectric (adapted from works by Lim et al. [42], Park et al. [43], Tsege et al. [53], Alluri et al. [55], Baek et al. [58]) and (**b**) piezoelectric polymers (adapted from works by Yaqoob et al. [39], Chen et al. [40], Jeong et al. [46], Shi et al. [51]).

While Park et al. reported the power density of ~7 mW/cm^3^ for p-NG made of thin BT transferred film without specifying the load resistance [17], Takahashi et al. demonstrated on another p-NG, based on BT film, the power of 2.3 µW at a single resistance of 1 MΩ, resulting in the calculated volumetric power density of 480 mW/cm^3^ based on reported p-NG area of 0.4 × 0.6 cm^2^ and BT film thickness of 200 nm [59]. There are also literature reports showing the output power as a function of applied external load resistance, as summarized in Figure 18. The left panel of Figure 18 includes composites of BT with non-piezoelectric additives and the right panel presents composites of BT with piezoelectric polymers. According to the plots, each p-NG has a load resistance at which its power output is maximal. Moreover, according to Table 6 and Figure 18, the composites without piezoelectric polymer can generate as high values for output power as ~40 μW at load resistance of ~500 MΩ for p-NGs based on BT NPs and NWs in a PDMS matrix [58]. All the other data, including composites with piezoelectric polymers (see Figure 18b), have shown lower values for output power, as well as lower optimum load resistance values.

**Figure 19:**
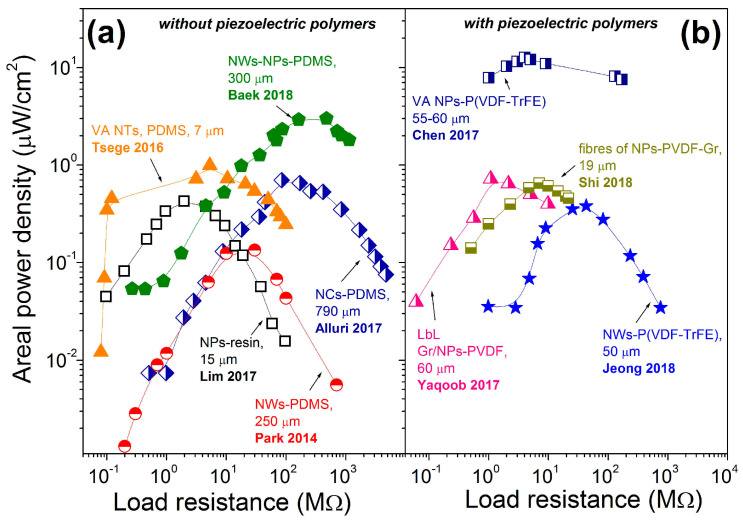
Areal power density as a function of external load resistance reported for flexible p-NGs based on composited, including BT nanomaterials, with (**a**) non-piezoelectric (adapted from works by Lim et al. [42], Park et al. [43], Tsege et al. [53], Alluri et al. [55], Baek et al. [58]) and (**b**) piezoelectric polymers (adapted from works by Yaqoob et al. [39], Chen et al. [40], Jeong et al. [46], Shi et al. [51]).

**Figure 20:**
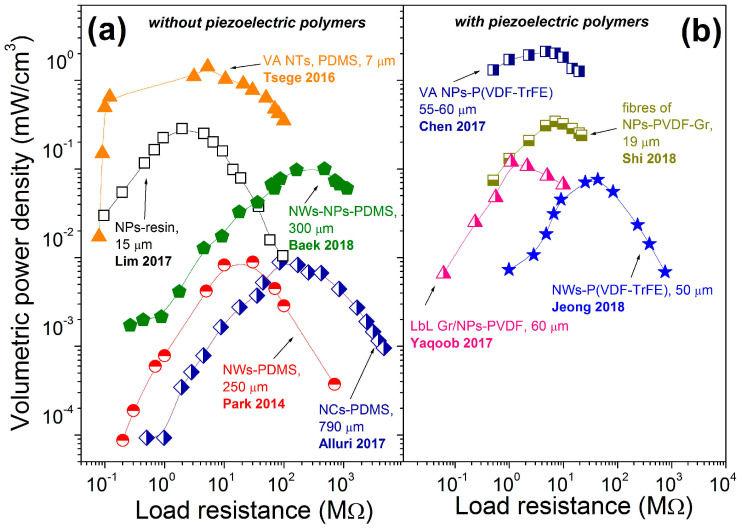
Volumetric power density as a function of external load resistance reported for flexible p-NGs based on composites, including BT nanomaterials, with (**a**) non-piezoelectric (adapted from works by Lim et al. [42], Park et al. [43], Tsege et al. [53], Alluri et al. [55], Baek et al. [58]) and (**b**) piezoelectric polymers (adapted from works by Yaqoob et al. [39], Chen et al. [40], Jeong et al. [46], Shi et al. [51]).

Another main comparable characteristic of piezoelectric nanogenerators for application is power density. Calculated from power, the power density is also dependent on the external load resistance, as shown in Figure 19 and Figure 20. According to Figure 19, the highest areal power density was obtained for the vertical micropillar based on BT NPs with piezoelectric polymer P(VDF-TrFE). The maximum areal output power density reached 12.7 μW/cm^2^, with a load resistance of 3.9 MΩ [40]. A similar areal power density of 12 μW/cm^2^ can be calculated for vertical BT NFs–PDMS p-NG with an area of 0.17 × 0.09 cm^2^, thickness of 670 μm and output power of 0.184 μW at a load resistance of 10 MΩ, reported by Yan et al. [49] and included in Table 6.

Regarding the reported and calculated volumetric power densities presented in Figure 20, high values can be obtained for p-NGs with a vertically oriented structure. A volumetric power density value of ~2.1 mW/cm^3^ was achieved for ~60 μm high vertical micropillars made of BT NPs–P(VDF–TrFE) by Chen et al. [40], and that of ~1.4 mW/cm^3^ was reported for ~7 μm high vertical BT NTs covered by PDMS by Tsege et al. [53]. However, the highest volumetric power density of 480 μW/cm^3^ can be calculated from a power value of 2.3 μW, reported by Takahashi et al. for p-NG prepared by transferring BT films, with very small thickness of 200 nm and area of 0.4 × 0.6 cm^2^ [59], as can also be seen from Table 6. All other composites have shown lower power density values by some orders of magnitude. Thus, high power density was obtained for p-NGs based on thin and small BT transferred films [59], or on BT structures with vertical geometry [40,53].

**Figure 21:**
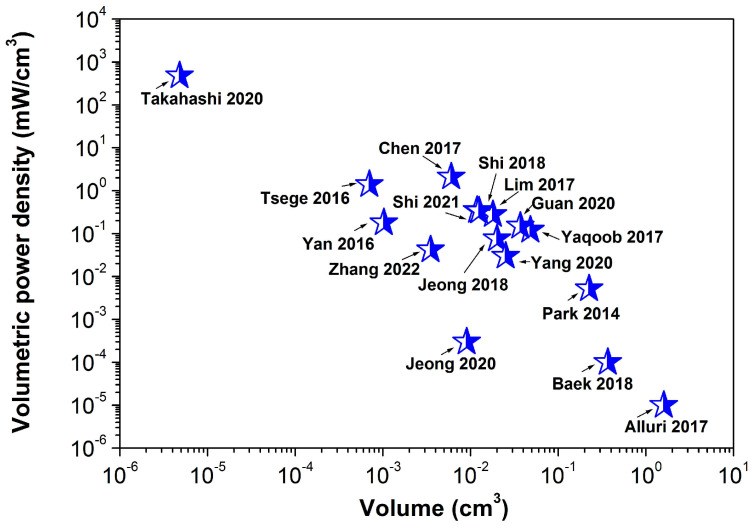
Dependency of the volumetric power density on the volume of the flexible BaTiO_3_-based piezoelectric composite layer (adapted from works by Yaqoob et al. [39], Chen et al. [40], Lim et al. [42], Park et al. [43], Jeong et al. [46], Yan et al. [49], Shi et al. [51], Tsege et al. [53], Alluri et al. [55], Baek et al. [58], Takahashi et al. [59], Jeong et al. [61], Zhang et al. [65], Shi et al. [66], Yang et al. [68], Guan et al. [69]).

In addition, to estimate the p-NG effectiveness, the maximum volumetric power density is plotted as a function of the piezoelectric composite layer volume in Figure 21, based on the available data for the reported areal size and thickness of the layer. The lower the volume, the higher the power density trend. The highest volumetric power density was obtained by Takahashi et al. for transferred BT film with a thickness of 200 nm [59].

## 7. Potential Application of BaTiO_3_-Based p-NGs

**Figure 22:**
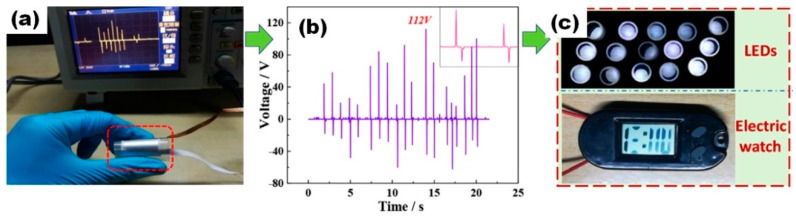
The optical image (**a**) and the output voltages (**b**) generated by a finger pressing-releasing process. A commercial electric watch and 15 LEDs driven by the converted electric energy from a finger pressing-releasing process (**c**). (Reproduced with permission of [51]. Copyright Elsevier, 2018).

As was reported in many articles and can be seen in Figure 22, p-NGs based on BaTiO_3_ nanomaterials can be used to power light emission diodes (LEDs) [51]. In the case shown in Figure 22, the electric energy is converted from a simple finger pressing-releasing process.

**Figure 23:**
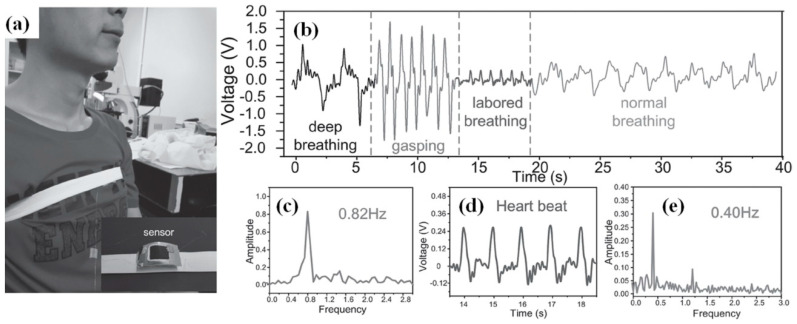
p-NG used for a highly sensitive wearable sensor for detecting human breathing motion (**a**). Output curves according to different breathing patterns (**b**). Fast Fourier transformation of waves of gasping (**c**) and normal breathing (**e**), respectively. An enlarged curve laboured breathing (**d**). (Reproduced with permission of [40]. Copyright Wiley, 2017).

However, usually p-NGs based on BaTiO_3_ nanomaterials have to be used as the energy source for small sensors such as air-pressure sensors in detecting the pressure on the noncontact-mode, proposed by Chen et al. [40]. Chen et al. also reported that such a p-NG device, fabricated using BT NPs–P(VDF–TrFE) composite, can be fixed on the chest to detect human breathing activity (see Figure 23a). Typically measured respiration signals for deep breathing, gasping, laboured breathing, and normal breathing modes are shown in Figure 23b–e. The curves of the output graphs closely follow the actual respiration cycle in both pitch and magnitude, which validates the effectiveness of a sensor used for reflecting the actual respiration cycle and different respiration modes. This can indicate that the highly sensitive, vertically well-aligned piezoelectrically enhanced nanocomposite micropillar array based nanogenerator can be applied as a wearable sensor for health monitoring [40].

**Figure 24:**
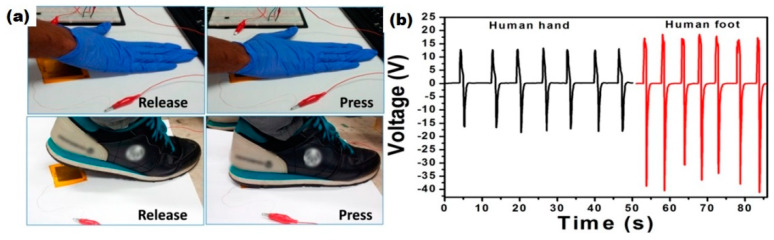
Real-time experimental demonstration using a composite p-NG device to harness low-frequency waste biomechanical energy. Photographs of human hand and foot release and press conditions acting on the composite p-NG device (**a**). Comparison of the open-circuit voltage when human hand and foot release/press force acted on the composite p-NG device (**b**). (Reprinted with permission from [55]. Copyright 2018, American Chemical Society).

Other popular contact pressure sensors based on p-NG were proposed by different research groups [55,68]. One of the sensors, reported by Alluri et al. and fabricated using p-NG based on BT NCs–PDMS composite, was sensitive to biomechanical energy from hand and foot stress and generated an output voltage of 55.9 V during foot stress (see Figure 24) [55]. Thus, BT NCs–PDMS composite p-NG used normal human physical motions and may be a reliable alternative and an unconventional energy harvesting approach.

**Figure 25:**
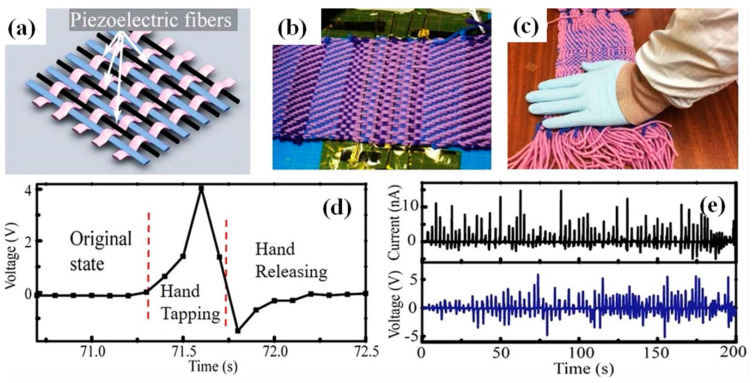
Potential applications for piezoelectric fibre generators. A cotton-based textile containing piezoelectric fibres woven using a loom (**a**,**b**). Electrical properties of the piezoelectric textile actuated by human hand tapping (**c**). Open-circuit voltages of the piezoelectric textile from hand tapping-releasing actions (**d**). Open-circuit voltages and short-circuit currents generated by the piezoelectric textile during repeated hand tap-release motions (**e**) [52].

Several other possible applications for p-NGs fabricated with fibres made of BT NPs-PVDF were proposed by Lu et al. [52]. They proposed integration of such piezoelectric fibres into large-area cotton textiles (see Figure 25). The obtained piezoelectric textiles can generate V_oc_ up to 5 V during repeated irregular deformations caused by human tapping-releasing actions (see Figure 25d,e).

**Figure 26:**
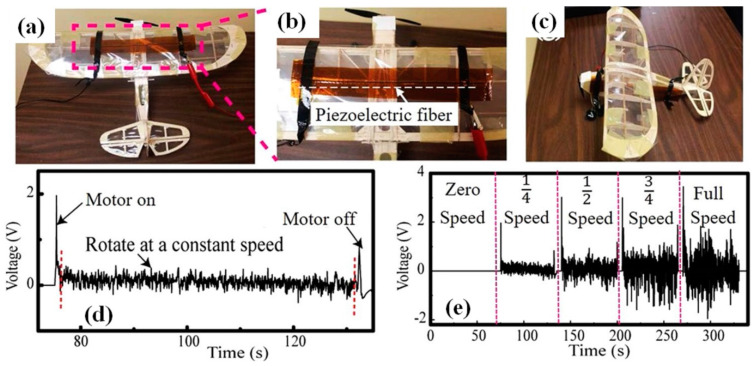
Piezoelectric fibres implanted on the airplane wing (**a**,**b**) and the airplane body (**c**). Open-circuit voltage generated by the piezoelectric fibres during rotation of the airplane propeller (**d**). Open-circuit voltages generated by the vibrations induced by the airplane motor operation with the motor speed set at zero, 1/4, 1/2, and 3/4 of its maximum (**e**) [52].

In the second prototype proposed by Lu et al., the piezoelectric fibre was glued onto the exterior of an airplane model (Figure 26) [52]. During the tests, the airplane model was fixed on a wooden table. As the airplane motor was turned on, the rotation of the airplane propeller resulted in irregular vibrations of the piezoelectric fibres, thus generating an electric signal (see Figure 26). The output voltages of the piezoelectric fibres are highly dependent on the rotation speed of the airplane motor. As the propeller rotation speed increased to the maximum, the open-circuit voltage of the piezoelectric fibre increased from 0 to 2 V [52].

Thus, since p-NGs can convert mechanical energy into electrical energy, one day they could replace toxic/chemical power cells. Moreover, flexible p-NGs on textiles create a possibility for use as energy transformation systems, together with online sensors for human health.

## 8. Conclusions

We have reviewed the recent advances in flexible generators based on piezoelectric BaTiO_3_ nanomaterials, highlighting these as a promising class of advanced energy harvesting devices. Although at a cost to the output signal, the combination of BT with polymers has enhanced mechanical stability, which is important for flexible p-NG applications. The optimal BT concentration can be found at around 20 wt.%. Poling is usually needed for the enhancement of the output performance of piezoelectric materials such as BaTiO_3_ and is obligatory for piezoelectric polymers such as PVDF (to obtain piezoelectric *β* phase). However, it can be avoided by the addition of conductive filler to BT-based nanocomposite, or in the case of the preparation of fibres by electrospinning. All forms of BT materials can be used in p-NG, although the highest values of power or power density were reported for thick composite films with BT nanoparticles fabricated in vertical orientation, or transferred thin BT films, respectively. Despite the increasing interest directed towards the development of small, lightweight, and flexible energy harvesting devices for advanced thin and wearable electronics, the output power performance of thick piezoelectric composite films was shown to be dependent on the strain/stress/angle, etc., in a similar way to that of thin composites.

The performance of flexible nanogenerators can still be enhanced by the BT geometry alignment between the generator electrodes, depending thus on the orientation of the BT component in the composite, similarly to that of other piezoelectric devices. Moreover, rational design can ensure reproducibility and better understanding of the structure–property relationships. Especially important is the fact that design and control of the texture and composition of the composites will possibly extend the potential use of BT nanowires, nanotubes, etc. Additional attention should be paid to such characteristics of BT nanomaterials as surface area, porosity, stretchability, durability, degradability, etc., which can be very important for practical use in flexible self-powered devices. Thus, future efforts should be focused on the geometry of the piezoelectric BT materials/composite layers, and control of the size, morphology, quantity, distribution and poling of functional components.

In addition, the packaging of the piezoelectric layer is very important and must be adequate. With continuous exploitation, it is believed that barium titanate composite materials will show a high enough power density to be realized in commercial piezoelectric nanogenerators. At the same time, the output performance of the prepared flexible p-NGs can be influenced by a connection with other devices requiring efficient power management. Thus, a lead-free piezoelectric self-powered nanogenerator based on BaTiO_3_ can be prepared and used in wearable electronics, different sensors/monitoring systems, and medical devices without negative effect on the environment. To conclude, new materials for mechanical energy harvesting/transformation, including new composites with BT, and a deeper understanding of the mechanism of charge transfer will lead to improvement in output characteristics and energy conversion efficiency.

## Figures and Tables

**Table 1:** Values of piezoelectric coefficients d_33_ and d_31_ for the most common piezoelectric materials [22,23,24,25].

Piezoelectric Coefficient, pm/V	PZT	BaTiO_3_	ZnO	AlN	PVFD	P(VDF-TrFE)	P(VDF-HFP)
|d_33_|	~60–593	~149–350	~5.9–44	~3.9–5.15	~25.8	~33.5	~32
|d_31_|	~120–274	~78	~5	~2	~22.4	~10.7	~43.1

**Table 2:** Comparison of the output voltage for flexible p-NGs with horizontally and vertically oriented BaTiO_3_ nanomaterials.

Work Layer	Work Area, cm^2^ (Thickness, μm)	Output Voltage, V	Working Mode	Ref.
Horizontal single BT NW covered by PDMS	- (≤0.35)	0.21	bending/stretching, 20 mm bending amplitude	[48]
Horizontal single fibre made of BT NWs-PVC	- (0.3)	0.9	bending/releasing	[44]
Horizontal BT NFs in PDMS	0.17 × 0.07 (650)	1.48	pressure 2 kPa	[49]
VA BT NFs in PDMS	0.17 × 0.09 (670)	2.67	pressure 2 kPa	[49]
VA BT NTs encapsulated by PDMS	1 × 1 (≤7)	10.6	bending/releasing, bending angle 70°	[53]
VA BT NPs-P(VDF-TrFE) covered by PDMS	1 (60)	13.2	force 50 N	[40]

**Table 3:** The main characteristics and parameters of flexible p-NGs based on BaTiO_3_ mixed with non-piezoelectric additives (ordered by the type of used BT nanomaterials).

Type of BaTiO_3_ (Size, nm)	Work Layer	Area, cm^2^ (Thickness, μm)	Bottom Layer|| Top Layer	Output Voltage, V	Output Current, μA	Type of Working Mode	Ref.
film	BT/SrTiO_3_ film	0.4 × 0.6 (0.2)	PET/ITO/PDMS|| PDMS/ITO/PET	1.5	-	vibration	[59]
film	BT film	0.82 (0.3)	Kapton/PDMS||PDMS	1	0.026	bending	[17]
Oriented NPs	BT NCs-PDMS	4 × 4 (-)	PET/Cu||Cu/PET	13	0.02	bending	[63]
NCr (≤100)	BT NCr-M13 virus-PDMS	2.5 × 2.5 (200)	PET/ITO/PDMS|| ITO/PET	6	0.3	bending	[57]
NCs (≤400)	BT NCs-PDMS	4.4 × 4.6 (790)	Kapton/Al||Al/Kapton	126.3	77.6	constant mechanical pressure ~0.001 MPa at fixed acceleration of 1 m/s^2^	[55]
NFs (∅354)	*vertical* BT NFs-PDMS	0.17 × 0.09 (670)	PET/ITO||ITO/PET	2.67	0.26	pressure 0.002 MPa	[49]
NFs (∅354)	BT NFs-PDMS	0.17 × 0.07 (650)	PET/ITO||ITO/PET	1.48	0.1	pressure 0.002 MPa	[49]
NFs (∅354)	BT NFs-PDMS	0.17 × 0.13 (430)	PET/ITO||ITO/PET	0.56	0.058	pressure 0.002 MPa	[49]
NTs (∅50)	*vertical* BT NTs-PDMS	1 × 1 (≤7)	PET/ITO|| PDMS/ITO/PET	10.6	1.1	bending/releasing, angle 70°	[53]
NTs (∅130)	*vertical* BT NTs-PDMS	2 × 3 (15)	Al||Ti/PET	1	0.02	bending	[61]
NTs (∅11)	BT NTs-PDMS	1 × 1 (300)	PS/Au/Cr||Au/Cr/PDMS	5.5	0.35	pressure 1 MPa	[54]
NWs (≤∅350)	BT NW-PDMS	(0.35)	PET/Ag||Ag/PDMS	0.21	0.0013	bending	[48]
NWs (∅156)	BT NWs-PDMS	3 × 3 (250)	PET/ITO/PDMS|| PDMS/ITO/PET	7	0.36	bending	[43]
NWs (∅300)	*fibres made of* BT NWs-PVC	(0.3)	PET/PDMS||Ag/Kapton	0.9	0.01	bending	[44]
NPs, NWs	BT NWs-BT NPs-PDMS	3.5 × 3.5 (300)	PET/ITO/PDMS|| PDMS/ITO/PET	60	1.1	5 mm displacement, rate 0.2 m/s	[58]
NPs (∅200)	BT NPs-PDMS	1 × 1 (200)	PET/Cu||ITO/PET	13.5	-	cantilever-type device, compressive force at 20 Hz	[37]
NPs (150)	BT NPs-resin	3 × 4 (~15)	Plastic/Ag||Ag/epoxy	7	2.5	strain 0.236%, rate 3.54%/s	[42]
NPs (20)	OA-BT NPs-PAA	1 (≤200)	Plastic/ITO||Al	1.8	0.7	force 51 N	[36]
NPs	BT NPs-chitosan	3 × 3 (160)	PET/Al||Al/PET	40.9	4.5	pressed/releasing	[62]
NPs (~100)	BT NPs-CNT-PDMS	5 × 7 (<300)	Kapton/Au-Cr|| PDMS/Au-Cr/Kapton	3.2	0.35	5 mm displacement, rate 0.2 m/s, strain 0.33%	[41]

**Table 4:** Main characteristics and parameters of the flexible p-NGs based on BaTiO_3_ mixed with piezoelectric polymers (ordered by type of BT nanomaterials used).

Type of BaTiO_3_ (Size, nm)	Work Layer	Area, cm^2^ (Thickness, µm)	Bottom Layer||Top Layer	Output Voltage, V	Output Current, μA	Working Mode	Ref.
NCs	BT NCs-PVDF	2.5 × 2.5 (~220)	PDMS/Al||Al/PDMS	7.99	1.01	pushing- releasing, force 11 N	[56]
NWs	BT NWs-PMMA-PVDF-TrFE	2.5 × 2.5 (20)	PET/Al||Al/PET	12.6	1.3	bending	[66]
NWs (∅150)	BT NWs-P(VDF-TrFE)	2 × 2 (50)	PET/ITO||Ti/Au	14	4	bending	[46]
NWs (∅170)	BT NWs-PVDF	-	In-Ag||Kapton	2	-	-	[67]
NWs (∅250)	BT NWs-PLA	1 × 4.5100 (100)	Stainless steel||Au	1.4	-	strain 0.35%	[47]
NWs (∅270)	fibres made of BT NWs-PVDF	3.5 × 3.5	Al||Al	0.7	-	pressure 0.04 MPa	[45]
NPs	BT NPs-PDA-PVDF	2.6 × 2.3 (36–42)	Al||Al	9.3	0.086	force 12 N	[68]
NPs	fibres made of BT NPs-PDA-PVDF-TrFE	2.5 × 2.5 (0.059)	Cu-Ni-fabric|| Cu-Ni-fabric	6	1.5	force 700 N at 3 Hz	[69]
NPs	BT-NPs-Cell-PVDF-TrFE	0.7 × 0.5 (100)	Au||Au	60	-	bending	[65]
NPs	fibres made of BT NPs-Gr-PVDF	2.5 × 2.5 (19)	PET/Al||Al/PET	11	-	strain 4 mm at 2 Hz	[51]
NPs	BT NPs-MWCNT-PVDF	3 × 1 (50)	PET/Al||Al/PET	4.4	0.66	force 2 N	[70]
NPs (50)	BT NPs-P(VDF-HFP)	0.785 (30)	Kapton/Al/PDMS|| PDMS/Al/Kapton	1.4	-	bending	[33]
NPs (100)	LbL Gr/BT NPs-PVDF	4 × 2 (60)	PET/ITO/Ag||Au/PET	10	2.5	force 2 N	[39]
NPs (100)	fibres made of BT NPs-P(VDF-TrFE)	0.785 (90)	PET/ITO||ITO/PET	12.46	3.65	force 20 N	[50]
NPs (100)	fibres made of BT NPs-P(VDF-TrFE)	0.785 (90)	PET/ITO||PDMS/ITO/PET	3.4	0.523	force 20 N	[50]
NPs (150)	fibre array of BT NPs-PVDF	1 × 1 (60)	PET/ITO||ITO/PET	35 (or 150)	0.6 (or 1.5)	pressure 1 MPa (or at 10 MPa)	[38]
NPs (200)	fibres made of BT NPs-PVDF	(100)	PS/Kapton/C-LDPE|| C-LDPE	8	0.05	bending	[52]
NPs (200)	BT NPs-P(VDF-HFP)	2.2 (50)	PI/Al/PDMS||Al/PI	110	22	normal to surface, pressure 0.23 MPa	[34]
NPs (200)	BT NPs-P(VDF-HFP)	2.2 (50)	PI/Al/PDMS||Al/PI	5	0.75	bending	[34]
NPs (200)	vertical array of BT NPs-P(VDF-TrFE)	1 (60)	Kapton/Au||MWCNT	13.2	0.3	force 50 N	[40]

**Table 5:** Reported output voltage before and after poling process and poling conditions of p-NGs (ordered by increasing applied field).

Applied Field, kV/cm	Time, h	Temperature, °C	Piezoelectric Composite	Output Voltage, V		Ref.
				Before Poling	After Poling	
5	24	80	BT NPs-PVDF fibre	~0	1	[52]
5	12	120	VA-BT NFs-PDMS	0.2	5	[49]
15	12	140	BT NWs-PDMS	~0	7	[40]
100	20	-	BT NPs-P(VDF-HFP)	0.5	1.2	[33]
100	20	150	BT NPs-CNT-PDMS	0.2	3.2	[41]
120	12	-	BT NPs-PDMS	9.2	13.5	[37]
150	1	100	n-Gr/BT NPs-PVDF	1.5	10	[39]
200	3	-	BT NPs-resin	~0	7	[42]
400	4	120	BT NWs-P(VDF-TrFE)	4	14	[46]

**Table 6:** Output power and power density of flexible p-NGs based on BaTiO_3,_ obtained with external load resistance, and their geometrical parameters (ordered by load resistance increase).

Work Layer	Load Resistance, MΩ	Area, cm^2^ (Thickness, μm)	Power, µW/Areal Power Density, µW/cm^2^/Volumetric Power Density, mW/cm^3^	Ref.
Gr/BT NPs-PVDF	1	4 × 2 (60)	5.8/0.73 */0.121 *	[39]
BT film-PDMS	1	0.4 × 0.6 (0.2)	2.3/9.6 */480 *	[59]
BT NPs-resin	2	3 × 4 (~15)	5/0.42/0.28 *	[42]
vertical array BT NPs-P(VDF-TrFE)	3.9	1 (60)	12.7 */12.7/2.1 *	[40]
fibres BT NPs-PDA-PVDF-TrFE	5	2.5 × 2.5 (59)	5.5 */0.878/0.15 *	[62]
vertical array BT NTs-PDMS	5.2	1 × 1 (≤7)	1 */1/1.4 *	[53]
fibres BT NPs-Gr-PVDF	6.9	2.5 × 2.5 (19)	4.1/0.66 */0.35 *	[51]
BT NWs-PMMA-PVDF-TrFE	7.2	2.5 × 2.5 (20)	4.25/0.68 */0.34 *	[66]
BT NWs-PVDF	9.3	-	-/1/-	[67]
vertical BT NFs-PDMS	10	0.17 × 0.09 (670)	0.184/12 */0.18 *	[49]
BT NWs-PDMS	20	3 × 3 (250)	1.2/0.13 */0.0052 *	[43]
BT NWs-P(VDF-TrFE)	30	2 × 2 (50)	1.5/0.38 */0.076 *	[46]
BT NCs-PDMS	35	4 × 4 (-)	2.6/0.16 */-	[63]
BT NPs-Cell-PVDF-TrFE	50	0.7 × 0.5 (100)	0.147 */0.42 */~0.042	[65]
BT NPs-PDA-PVDF	70	2.6 × 2.3 (42)	0.73 */0.122/0.03 *	[68]
vertical array BT NPs-PDMS	100	2 × 3 (15)	0.003/0.0005/0.0003 *	[61]
BT NCs-PDMS	100	4.4 × 4.6 (790)	16 */0.8/0.00001 *	[55]
BT NWs-BT NPs-PDMS	500	3.5 × 3.5 (300)	~40/3.3 */0.0001 *	[58]

* Calculated value based on the reported p-NG geometrical parameters.

## Data Availability

The data presented in this study are available on request from the corresponding author.

## Glossary

BT	barium titanate
BT NF-*H*	barium titanate nanofibres horizontal
BT NF-*R*	barium titanate nanofibres random
BT NF-*V*	barium titanate nanofibres vertical
C-LDPE	carbon-impregnated low density polyethylene
CNT	carbon nanotubes
DMF	dimethylformamide
f-PEH	flexible piezoelectric energy harvester
I_sc_	short-circuit current
ITO	indium tin oxide
LbL	layer-by-layer
LED	light emission diode
MWCNT	multi wall carbon nanotubes
NCs	nanocubes
NCr	nanocrystals
Gr	graphene
NFs	nanofibres
NPs	nanoparticles
NTs	nanotubes
NWs	nanowires
OA	oleic acid
PAA	poly(acrylic acid)
PDA	polydopamine
PDMS	polydimethylsiloxane
PEH	piezoelectric energy harvester
PET	polyethylene terephthalate
PI	polyimide
PLA	polylactic acid
p-NG	piezo nanogenerator
PS	polystyrene
PVC	polyvinyl chloride
PVDF	polyvinylidene fluoride
P(VDF-HFP)	poly(vinylidene fluoride-co-hexafluoropropylene)
P(VDF-TrFE)	poly(vinylidene fluoride-co-trifluoroethylene)
PZT	lead zirconate titanate
RGO	reduced graphene oxide
SEM	scanning electron microscopy
SPs	spherical nanoparticles
V_oc_	open-circuit voltage

## Appendix A

**Table A1:** Reported details of poling process of flexible p-NGs based on BaTiO_3_ nanomaterials (ordered by increasing applied electric field).

Applied Field or Voltage	Time, h	Temperature, °C	Work Layer	The Highest Output Voltage, V	Ref.
30 kV/cm	3	100	fibres made of BT NWs-PVC	0.9	[44]
50 kV/cm	12	120	Random BT NFs-PDMS	0.56	[49]
50 kV/cm	12	120	Horizontal BT NFs in PDMS	1.48	[49]
50 kV/cm	12	120	VA BT NFs in PDMS	2.67	[49]
80 kV/cm	12	*rt*	BT NTs-PDMS	5.5	[54]
100 kV/cm	1	rt	horizontal BT NW covered by PDMS	0.21	[48]
100 kV/cm	12	90	BT NWs-PLA	0.49	[47]
100 kV/cm	15	140	MIM ribbons of BT nanofilm	1	[17]
100 kV/cm	20	100	BT NPs-P(VDF-HFP)	1.4	[33]
100 kV/cm	20	100	BT NPs-P(VDF-HFP)	110	[34]
100 kV/cm	20	150	BT NPs-(CNT or RGO)-PDMS	3.2	[41]
100 kV/cm	20	100	BT NPs-P(VDF-HFP)	75	[35]
±120 kV/cm	12	rt	BT NPs-PDMS	13.5	[37]
150 kV/cm	1	100	LbL Gr/BT NPs-PVDF	10	[39]
200 kV/cm	3	rt	BT NPs-resin	7	[42]
400 (100 *) kV/cm	4 (2 *)	120 (50 *)	BT NWs-P(VDF-TrFE)	14	[46]
500 kV/cm	-	-	VA BT NPs-P(VDF-TrFE)	13.2	[40]
1 kV	12	120	BT NWs-BT NPs-PDMS	60	[58]
1.5 kV	12	140	BT NWs-PDMS	7	[43]
2 kV	8	*rt*	BT NPs-PVDF	150	[38]
2 kV	12	130	BT NCr-M13 virus-PDMS	6	[57]
5 kV	24	80	fibres made of BT NPs-PVDF	8	[52]
8 kV	24	*rt*	BT NCs-PDMS	126.3	[55]
8 kV	24	*rt*	BT NCs-PVDF	11.9	[56]
15 kV	4	*rt*	VA of BT NTs, PDMS	10.6	[53]

* In reverse direction. rt-room temperature.
